# The Analysis of the Influence of Threshold on the Dynamic Contact Process of a Fabricated Vertically Driven MEMS Inertial Switch

**DOI:** 10.3390/mi10110791

**Published:** 2019-11-18

**Authors:** Wenguo Chen, Rui Wang, Huiying Wang, Dejian Kong, Shulei Sun

**Affiliations:** 1 The College of Information Engineering, Qujing Normal University, Qujing 655000, China; chenwenguo@163.com (W.C.); wangrui100865@163.com (R.W.); kingwhy2008@163.com (H.W.); 2 The College of Mechanical Engineering, Guizhou Institute of Technology, Guiyang 550003, China; bobsunstuy@gmail.com

**Keywords:** MEMS inertial switch, surface micromachining, dynamic contact process, threshold

## Abstract

In this work, to evaluate the influence of the threshold on the dynamic contact process, five models (number 1, 2, 3, 4, 5) with different thresholds were proposed and fabricated with surface micromachining technology. The contact time and response time were used to characterize the dynamic contact performance. The dynamic contact processes of the inertial switches with gradually increasing thresholds were researched using analytical, simulation, and experimental methods. The basic working principle analysis of the inertial switch shows that the contact time of the inertial switch with a low-g value can be extended by using a simply supported beam as the fixed electrode, but the high-G inertial needs more elasticity for fixed electrode. The simulation results indicate that the response time and contact time decrease with the increment in the designed threshold. Prototypes were tested using a dropping hammer system, and the test result indicates that the contact time of the inertial switch with a fixed electrode of the simply supported beam is about 15 and 5 μs when the threshold is about 280 and 580 g, respectively. Meanwhile, the contact time can be extended to 100 μs for the inertial switch using a spring as the fixed electrode when the threshold is about 280 and 580 g. These test results not only prove that the spring fixed electrode can effectively extend the contact time, but also prove that the style of the fixed electrode is the deciding factor affecting the contact time of the high-G inertial switch.

## 1. Introduction

The MEMS inertial switch, as a passive device, is widely used in Internet of Things (IoT) systems, particularly in areas where the power supply is limited, which has become a research hotspot in recent years due to its superior performance [1,2,3,4]. Especially in recent years with the rapid development of the Internet of Things, the MEMS inertial switch has been more widely used. For example, a longitudinally driven inertial switch was proposed for IoT applications by Qiu et al. [5]. Ongkodjojo and Tay reported a G-switch comprising an elastic beam for healthcare applications [6]. A shock sensor with a latching structure was proposed for health monitoring by Whitley et al. [7]. Kuo et al. presented an inertial switch employing stimuli-sensitive hydrogel integrated with a passive inductor/capacitor (LC) resonator [8], which was an active device. In IoT systems, as a part of intelligent information systems, the overall performance is affected by the individual sensors. The main parameters used to evaluate the performance of inertial switches include threshold acceleration (*a_th_*), contact time (*t_co_*), and response time (*t_re_*). Contact time is an important factor affecting the performance of inertial switches, where short contact times of the output signal will lead to signal processing difficulties. To identify an output signal with a short contact time, the precision and sensitivity of the signal identification system must be improved. However, the signal identification system with a high sensitivity may recognize the interference signal with a narrow pulse width as the output signal, which will result in a poor overall judgment of the system. On account of this, the extended contact time is beneficial to improve the reliability of the system.

To prolong the contact time, Yang, Ding, and Cai, et al. proposed a vertically sensitive inertial switch and extended the contact time by designing an elastic point on the mass block [9,10,11,12,13]. The teste results showed that the designed fixed electrode can effectively prolong the contact time. However, an interesting phenomenon was discovered in our experiment: The contact time became very short when the design threshold (*a_th_*) exceeded 300 g. In addition, in the previous experimental results [11], we confirmed that the simply supported cross beams used as a fixed electrode did have the effect of extending the contact time compared to the rigid body without elasticity. Therefore, why did the simply supported cross beams lose the effect of extending the contact time at a higher design threshold? To answer the question, a series of models with different thresholds were proposed in this work, and the relationship between threshold, contact time, and response time was analyzed. The results explained why the simply supported beam could not extend the contact time in the application of the high-threshold inertial switch. The influence rules of the threshold acceleration on the contact time and response time were analyzed, and the dynamic contact processes of different designed models were provided. Based on the influence rules, a high-G (e.g., *a_th_* ≥ 500 g) design scheme, which can effectively prolong contact time, was proposed. Prototypes of designed inertial switches were fabricated and tested, and the test results indicated that the contact time could be prolonged for the inertial switch with a lower design threshold (e.g., *a_th_* = 20 g) owing to the small elastic resilience of the spring. On the contrary, it is difficult to prolong the contact time if the same design is used when the design threshold goes up (e.g., *a_th_* ≥ 500 g). The high-G inertial switch requires a higher elasticity of the fixed electrode. Meanwhile, the test result indicated that the proposed inertial switch with the spring fixed electrode could prolong the contact time when the design threshold was 500 g.

## 2. Designing Schemes

**Figure 1:**
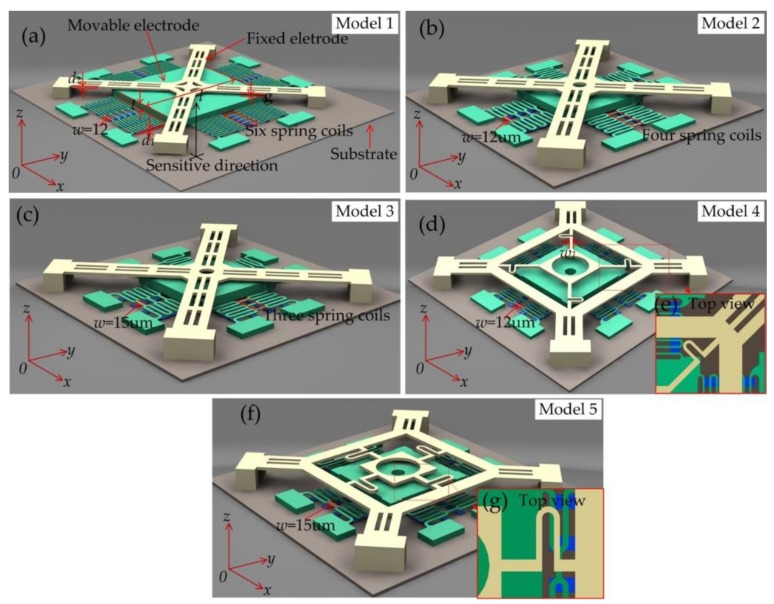
Design models with different thresholds. (**a**) Movable electrode with six spring coils and fixed electrode with perforated cantilever, (**b**) four spring coils, (**c**) three spring coils, (**d**) movable electrode with three spring coils and a fixed electrode constituted by springs, (**e**) top view of the location of the fixed electrode shown in (**d**), (**f**) additional model with the spring fixed electrode, (**g**) top view of the fixed electrode shown in (**f**).

In order to investigate the dynamic contact process of the inertial switch with different threshold accelerations, five designed models have been proposed, numbered 1, 2, 3, 4, and 5, as shown in Figure 1. These models were selected with similar architectures using a micro-lamellar structure. The main structures of the designed models consist of three parts: Substrate, movable electrode, and fixed electrode. The quartz glass was selected as the substrate, and a seriously raised metal bar was electroplated on the substrate to reduce the damping effect [14,15,16]. The proof mass was suspended by four groups of serpentine springs as a movable electrode. The fixed electrode was placed above the proof mass. When acceleration was applied to the inertial switch in the sensitive direction (z-direction), the movable electrode moves rapidly towards to the fixed electrode and the external circuit closed while the acceleration reached the threshold. In this experimental scheme, the threshold acceleration is controlled by the type of movable electrode, which is mainly determined by the stiffness of the spring. The threshold of the inertial switch increased with the increment in the movable electrode’s stiffness, while the other conditions remain unchanged. Therefore, the quantity of suspended springs and the mass of movable electrodes are the primary variables used to change the threshold in this work. The stiffness of the fixed electrode can be changed by modifying the structural shape. The movable electrodes are shown in Figure 1a–c and consist of the proof mass and six, four, and three springs, respectively. The fixed electrode shown in Figure 1a is similar to those in Figure 1b,c, the fixed electrode consists of simply supported cross beams, and the elasticity of the electrode is mainly derived from the ductility of the metal material. The fixed electrodes shown in Figure 1d,e consist of a cross-shaped structure consisting of four springs. The top views of the spring electrode are shown in Figure 1e,g, and the location of the spring and the proof mass were set to the same vertical plane, which can effectively serve as the elastic function of the spring. The main geometric parameters of the design model are shown as Table 1. The symbols of *t* and *l* are the thickness and length of the proof mass, respectively. The gap between the electrodes is expressed by *g*, *w* and *w*_1_ are the widths, and *d*_1_ and *d*_2_ are the thicknesses of the suspension spring of the movable and fixed electrode, respectively. 

## 3. Physical Model and Working Principle

**Figure 2:**
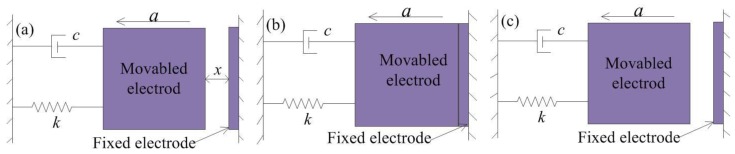
Rigid contact process of inertial switch. (**a**) the acceleration is applied to the inertial switch, (**b**) the movable electrode contacts with fixed electrode, (**c**) the movable electrode returns to original position.

The MEMS inertial switch is an inertial element that can run the external circuit through driving acceleration and is both a sensor and an actuator. The mechanical inertial switch consists of two parts: A movable electrode and fixed electrode. When the inertial switch is impacted by acceleration exceeding the threshold value, the movable electrode moves quickly and contacts with the fixed electrode, and the external circuit is switched on instantaneously. The basic idealized physical model of the model is shown in Figure 2 [17,18], where *c* is the damping coefficient, *a* is the acceleration, and *k* is the elastic coefficient of the suspension spring. The typical contact process is often designed as a rigid contact process due to the limitation of technology conditions. The rigid contact process can be described as follows:

(a) The acceleration load is applied to the inertial switch in the sensitive direction, and the proof mass moves toward the fixed electrode.
(b) The proof mass contacts with the fixed electrode when the acceleration reaches the threshold, and the contact surfaces are positioned in the same plane.
(c) The proof mass bounces off the fixed electrode back to the initial position owing to the elastic force of the fixed electrode and spring k.

During this contact process, the stiffness of the movable electrode and the mass of the proof mass are the main factors affecting the contact time, which are also key factors affecting the threshold. The contact time of this rigid contact process is very short due to the rigidity of the fixed electrode approaching infinity, and the contact time is often less than 5 μs [19].

When the displacement of the movable electrode reaches *x*_0_, the elastic restoring force (*f* = *kx*_0_) equals the force (*F = ma_t_*) attached to acceleration while the elasticity of the fixed electrode is ignored, which can be described as Equation (1).

(1) $$kx_{0}=ma_{t},$$

where *k* is the elastic coefficient of movable springs, *x*_0_ is the displacement of the proof mass, *m* is the mass of the movable electrode, and *a_t_* is the threshold acceleration.

In this case, because of the short duration of the load on the electrodes, the response of the movable electrode can be evaluated by impulse (*I*) described as Equation (2) [20].

(2) $$m\Delta \dot{\upsilon }=\int_{0}^{t_{1}}\left[a(t)-kx_{0}\right]dt,$$

where a(t) is the applied load, $m\Delta \dot{\upsilon }$ is the momentum of the movable electrode, and $t_{1}$ is the pulse width of the applied load. One can obtain from Equation (2) that the time (dt) increases while the mass of the proof mass (m) is added, and decreases while the threshold acceleration load (a(t)) is increased.

Then, two conclusions can be obtained when Equation (2) is introduced to this model, which is described as follows:

(1) The larger the mass (*m*), the larger the momentum (mΔv), and it will take longer to change the motion state of the movable electrode. When the inertial switch is shocked by applied acceleration, it will take a long time for the mass to change from the static state to the moving state, which will result in a longer response time. When the displacement of the proof mass reaches *x*_0_, the time taken to move the proof mass in the opposite direction is the contact time, which can draw the conclusion that the contact time increases with the mass of the proof mass.
(2) The smaller the load (a(t)), the longer it takes to change the state of motion. One can obtain the conclusion that the contact time decreases with the applied load.

In addition, the load applied to the inertial switch is a half-sine wave acceleration in practical application, and the response time (*t_re_*) and contact time (*t_con_*) are affected by the pulse width ($t_{1}$) of the applied acceleration. Generally, the pulse width ($t_{1}$) of the acceleration with a lower threshold is larger. In other words, the overall performance of the proposed device is determined by the frequency response characteristic of the movable electrode. For the dynamic response of the inertial switch, the response process of the device is determined by the inherent frequency ($\omega_{0}$) of the movable electrode when the applied load has been provided. Thus, response time (*t_re_*) is an extraordinary factor for evaluating the performance of the contact process, and the contact time increases with the response time. An inertial switch with a high performance requires a short response time and long contact time, but there is a contradiction between these two parameters. When the inertial switch is shocked by a half-sine acceleration, the response time can be divided into two parts: One part is the time required for the movable electrode to change from the static state to the moving state, and the other part is the pulse duration of the applied load. As only the effect of the acceleration threshold on the contact time is considered here, the duration of the pulse is ignored. To analyze the minimum response time of the inertial switch, the acceleration (*a* = *a*_0_) with a step change is used to qualitatively evaluate the response time. When the damping is ignored, the motion equation of acceleration (*a*_0_) can be written as [20]

(3) $$\frac{d^{2}x}{dt^{2}}+\omega_{0}^{2}x=a_{0},$$

where $a_{0}$ is the maximal acceleration, $\omega_{0}$ is the inherent frequency of the movable electrode, and x is the gap between electrodes, which is also the displacement of the movable electrode.

By solving Equation (3), the general solution is

(4) $$x(t)=c_{1}\sin\omega_{0}t+c_{2}\cos\omega_{0}t+\frac{a_{0}}{\omega_{0}^{2}},$$

where *c*_1_ and *c*_2_ are constant.

By applying the initial conditions of displacement and velocity (x(0)=0,v(0)=0), the dynamic equation of displacement can be written as

(5) $$x=\frac{a_{0}}{\omega_{0}^{2}}(1-\cos\omega_{0}t)=\frac{2a_{0}}{\omega_{0}^{2}}\sin^{2}(\frac{\omega_{0}t}{2}).$$

From Equation (5), the response time of the system can be written as

(6) $$t_{re}=\frac{1}{\omega_{0}}\arccos(1-\frac{x_{0}\omega_{0}^{2}}{a_{0}}).$$

**Figure 3:**
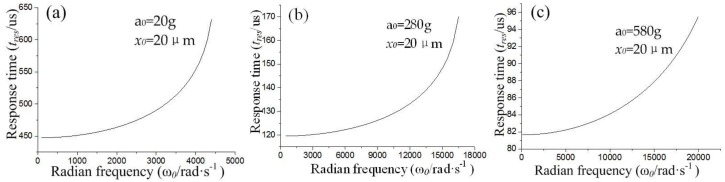
Relation curves of response time (*t_re_*) between inherent frequency ($\omega_{0}$). (**a**) the inertial switch with threshold is 20 g, (**b**) 280 g, (**c**) 580 g.

According to Equation (6), the response time is determined by the inherent frequency ($\omega_{0}$) and acceleration peak value (*a*_0_), and the gap (*x*_0_) between electrodes is a constant. The relation curves between the response time (*t_re_*) and acceleration (*a*_0_), and inherent frequency ($\omega_{0}$), are shown in Figure 3.

Figure 3 indicates that the response time (*t_re_*) increases with the threshold acceleration (*a*_0_). The minimum response times are about 450, 120, and 82 μs when the threshold is about 20, 280, and 580 g, respectively. The contact time decreases with the response time without considering other influencing factors. Thus, we have another conclusion that the contact time (*t_con_*) decreases with the increase in threshold acceleration.

In the structure of the inertial switch, with the increase in mass of the movable electrode, lowering the force of the springs will result in a lower threshold. The above conclusions indicate that the inertial switch with a low threshold value has a long contact time. On the contrary, the contact time decreases as the threshold is increased. In addition, although the above conclusions reveal the influence of the design threshold on contact time, it is impossible to realize an ideal contact state with the electrodes in practice, so it is necessary to investigate the contact process under certain overload conditions. Therefore, the elasticity of the fixed electrode is an important factor that affects the contact time when the overload acceleration is applied to the device. For a low-G designed inertial switch, the requirement of elasticity of the fixed electrode is lower than a high-G switch because the overloading used to keep the switch in the on state is small. The fixed electrode with a high rigidity causes the proof mass to quickly return to the equilibrium position, which will result in a very short contact process for the high-G inertial switch. Thus, improving the elasticity of the fixed electrode is an effective way to extend the contact time. 

**Figure 4:**
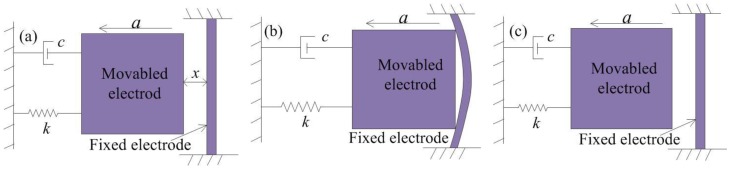
Contact process of inertial switch with simply supported beam. (**a**) the acceleration is applied to inertial switch with fixed electrode of simply supported beam, (**b**) the fixed electrode of the simply supported beam has elastic deformation due to the inertia impact of movable electrode, (**c**) the movable electrode returns to its original position.

In this paper, a simply supported beam and spring-connected cross beam were proposed as the fixed electrode. The contact process of the inertial switch with the simply supported beam is shown in Figure 4a. Figure 4b shows that the movable electrode contacts with the fixed electrode when the applied acceleration reaches the threshold. In addition, the simply supported beam bends when it is impacted by the inertia of the mass block. In Figure 4c, the movable electrode returns to the initial position due to the elastic force of the spring and simply supported beam. In this contact, the contact time is prolonged owing to the bending of the simply supported beam. However, the effect of extending the contact time is very small due to the large stiffness of the simply supported beam.

**Figure 5:**
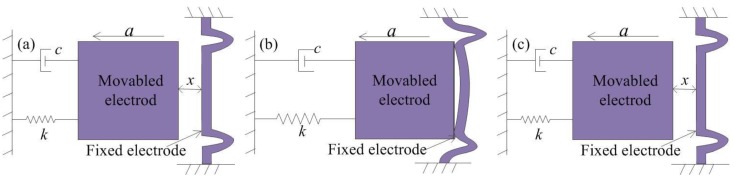
Contact process of inertial switch with spring-connected cross beam. (**a**) the acceleration is applied to inertial switch with fixed electrode of spring, (**b**) the fixed electrode of spring has elastic deformation due to the inertia impact of movable electrode, (**c**) the movable electrode returns to its original position.

The contact process of the inertial switch with the spring-connected cross beam is shown in Figure 5. Figure 5b shows that the elastic deformation of the fixed electrode comprising springs occurred by the impact of the movable electrode, and the movable electrode and fixed electrode move cooperatively for a short time in the same direction. During the whole impact process, the movable and the fixed electrode are always in contact due to the low stiffness of the spring. Figure 3c shows that the movable electrode and fixed electrode return to the initial position under the elastic restoring force. Compared to the simply supported beams, this contact process indicates that the contact time is prolonged owing to the elastic deformation of the suspended spring.

## 4. Dynamic Simulation Analysis

**Figure 6:**
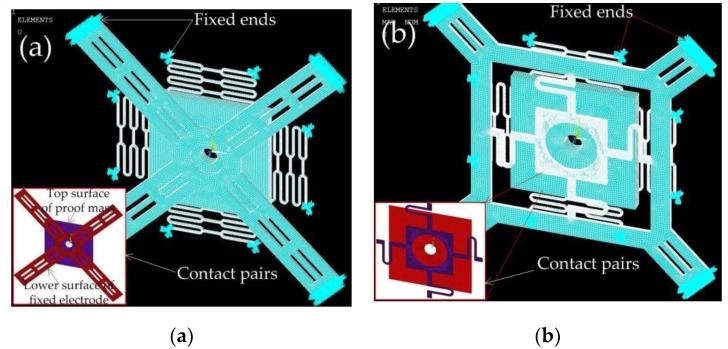
Finite element models of inertial switch with fixed electrode of (**a**) simply supported beam and (**b**) suspension spring.

**Figure 7:**
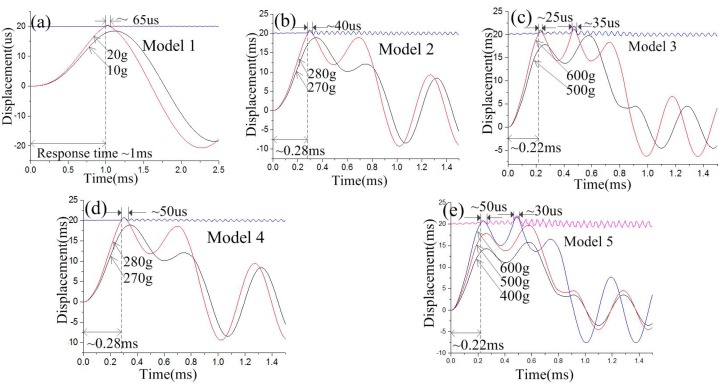
Dynamic response curves of inertial switch with different thresholds, and the thresholds of the designed models are (**a**) 20, (**b**) 280, (**c**) 600, (**d**) 280, and (**e**) 600 g.

To investigate the contact process of the inertial switches with different fixed electrodes, the designed structures are modeled and simulated by ANSYS software. The finite element model of the inertial switch with a fixed electrode using the simply supported beam and suspension spring is shown in Figure 6a,b, respectively. The lower surface of the fixed electrode and the upper surface of the movable mass block are defined as a pair of contact surfaces. The end surfaces of the fixed electrode and movable electrode are defined as fixed surfaces. The nickel (Ni) electroplated by surface micromachining technology is selected as the structure, and the material parameters of Young’s modulus and the density are chosen as 165 GPa and 8.96 g∙cm^−3^, respectively [21]. The half-sinusoidal acceleration load with a pulse width of one millisecond is applied to the finite element models. The dynamic response curves of the inertial switch with different threshold accelerations are shown in Figure 7a–e. The dynamic response process indicates that the designed thresholds of models 1, 2, 3, 4, and 5 are 20, 280, 600, 280, and 600 g, respectively. The contact time is about 65, 40, 25, 50, and 50 μs, respectively. The response times are about 1, 0.28, 0.22, 0.28, and 0.22 ms, respectively. The response times from the simulation results are much more than those from the theoretical analysis because the duration of the applied load is ignored when the initial conditions of displacement and velocity (x(0)=0,v(0)=0) are applied to Equation (5) to reveal the minimum response time. The contact time decreases from 65 to 35 μs, and the contact time decreases from 1 to 0.22 ms, when the threshold increases from 20 to 600 g. As the response time is mainly determined by the inherent frequency ($\omega_{0}$) of the movable electrode, the response time of the device remains unchanged when the structure of the fixed electrode is replaced by the spring. When the fixed electrode with the simply supported beam is replaced by the suspension spring, the contact time is prolonged from 40 to 50 μs and 25 to 50 μs when the thresholds are 280 and 600 g, respectively.

**Figure 8:**
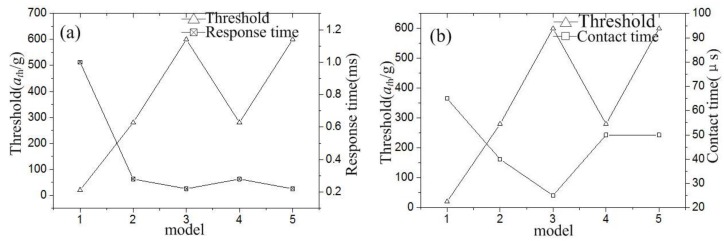
(**a**) Relationship between response time and threshold. (**b**) Relationship between contact time and threshold.

The relationship between the response time and threshold is shown in Figure 8a, which indicates that the response time decreases with increasing threshold. In addition, the response time remains unchanged even when the fixed electrode design is changed. The relationship between the contact time and threshold is shown in Figure 8b, which indicates that the contact time decreases as the threshold increased. However, the contact time is prolonged to 50 μs with the fixed electrode with the simply supported beam instead of the suspension spring. The contact time holds for 50 μs when the threshold acceleration increases from 280 to 600 g. This is because the contact of the inertial switch with a high G value is realized under overload acceleration. When the design threshold is 280 and 600 g, the overload acceleration causes the spring to have the same displacement, so they have the same contact time, which draws the conclusion that the contact time is decided by the stiffness of the fixed electrode for the high-G (e.g., ≥280 g) inertial switch.

**Figure 9:**
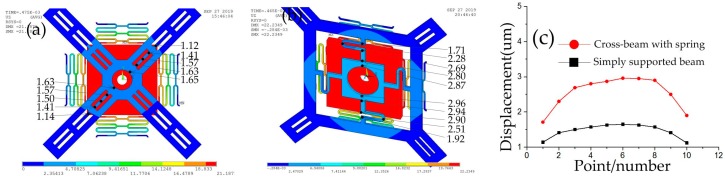
(**a**) Maximum displacement of model 3 with 600 g acceleration. (**b**) Maximum displacement of model 5 with 600 g acceleration. (**c**) Vibration tracks of fixed electrodes.

When the threshold acceleration of 600 g is applied to model 3 and 5, the maximum displacement of the inertial switch with the simply supported beam and suspension spring is obtained using ANSYS software, as shown in Figure 9a,b. By querying the grid data, the maximum displacement of the simply supported beam is about 1.65 μm, as shown in Figure 9a. The maximum displacement of the suspension spring is about 2.96 μm, as shown in Figure 9b. By comparing Figure 9a,b, it is found that the displacement of the spring fixed electrode is larger than that of the simply supported beam. The vibration tracks of the simply supported beam and cross beam with the spring are shown in Figure 9c. The stiffness of the fixed electrodes in model 3 and model 5 was calculated by ANSYS software as ~851 and ~675 n/m, respectively. Compared to the simply supported beam, the fixed electrode consisting of springs can more effectively extend the contact time due to a greater elastic displacement.

Through the above simulation analysis, the relationship between the contact time, response time, and the design threshold of the inertial switch is revealed, and a solution to prolong the contact time of the high-G-value inertial switch is proposed through the spring fixed electrode structure.

## 5. Fabrication Prototype

**Figure 10:**
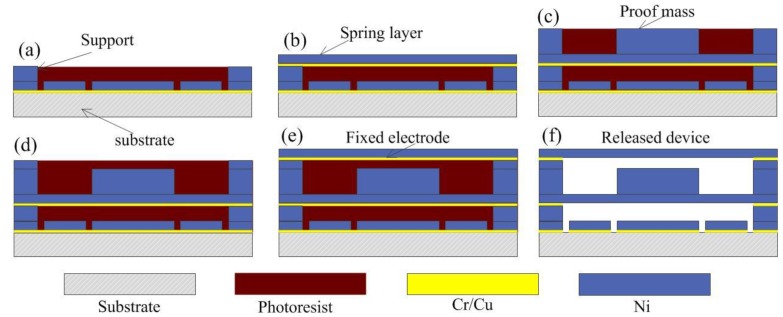
Schematic diagram of primary fabrication process.

The prototypes are fabricated by surface micromachining technology, all devices are manufactured on one wafer with the same set of masks, and the main preparation technique method includes the magnetron sputtering coating process, micro electroforming, and SU-8 photolithography techniques. The critical process steps are shown in Figure 10 and described as follows:

(a) After sputtering the Cr/Cu seed layer on the substrate, the support and strip structure were electroplated by a photolithography pattern, and the height of the supporting structure was higher than that of the strip.
(b) The spring for hanging the movable electrode was fabricated above the strip pattern, and the bottom layer of the mass block was also prepared.
(c) The sensitive mass was prepared by multiple electroplating processes until the thickness of the mass blocks reached the designed value.
(d) The fixed electrode support was fabricated higher than the sensitive mass.
(e) The Cr/Cu seed layer was sputtered on the fixed electrode support, and the fixed electrode was fabricated above the sensitive mass.
(f) The photoresist and metal seed layer were removed selectively. The completed device structure was obtained.

**Figure 11:**
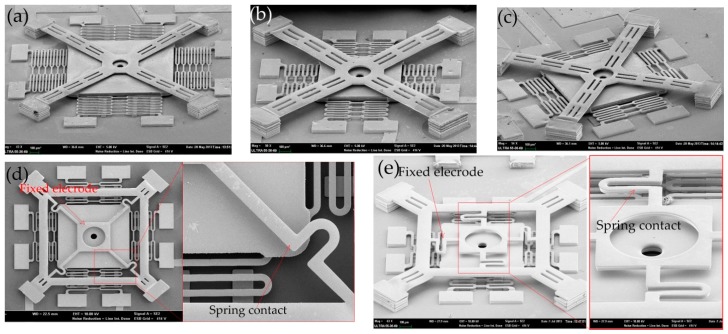
SEM images of prototype devices. (**a**–**c**) Inertial switch using simply supported beams as the fixed electrode, (**d**,**e**) inertial switch using a spring as the fixed electrode.

The designed models 1, 2, 3, 4, and 5 were fabricated by surface micromachining technology, as shown in Figure 11a–e. All the prototypes with the complete structure are shown in Figure 11.

## 6. Test and Analysis

**Figure 12:**
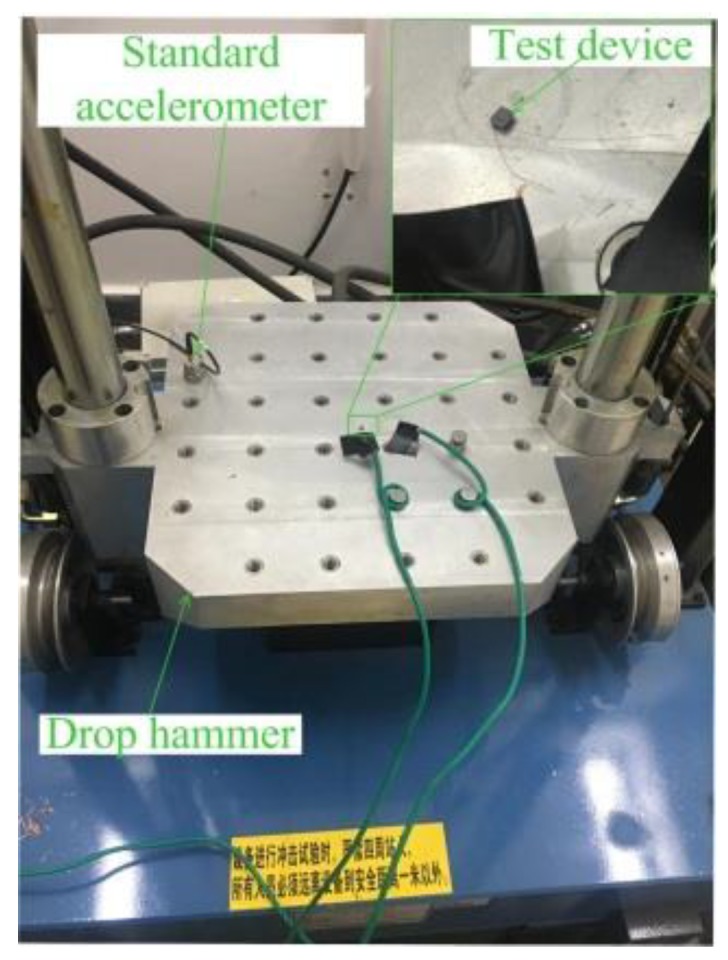
Dropping hammer system.

**Figure 13:**
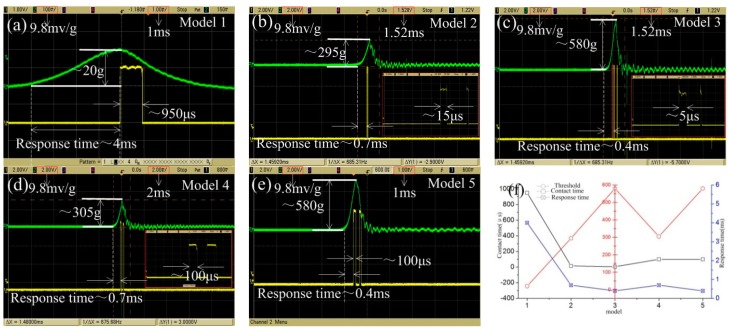
Tested results of prototypes with different thresholds. (**a**) 20, (**b**) 295, (**c**) 580, (**d**) 305, and (**e**) 580 g, and (**f**) curves of the threshold, contact time, and response time.

The fabricated inertial switch was tested by a hammer dropping experiment. The thresholds of five devices were tested by the drop hammer. The packaged device and standard accelerometer were fixed on the platform, as shown in Figure 12. The thresholds of the fabricated prototypes were measured by the dropping hammer, and the preset height of the hammer increases gradually from low to high, which means that the deceleration value applied to the test device increases gradually. When the acceleration reaches the threshold value of the testing device, the inertial switch will be switched on, and the trigger signal and acceleration curves will output to the oscilloscope. The test results of the devices with different thresholds are shown in Figure 13.

The test thresholds of model 1, 2, 3, 4, and 5 are 20, 295, 580, 305, and 580 g, respectively. The test results indicate that the contact time decreases from 950 to 5 μs when the threshold acceleration increases from 20 to 580 g. The test results of the prototypes with the spring fixed electrode shown in Figure 13d,e show that, compared to model 2 and 3, the contact time is prolonged to 100 μs when the thresholds are 305 and 580 g, which indicates that the contact time can be prolonged due to the elastic fixed electrode. This is because the contact time mainly depends on the stiffness of the fixed electrode under the overload acceleration. Figure 13f shows the relation curves of the contact time and response time with increasing threshold. The curves indicate that the contact time and response time decrease with the increasing threshold; meanwhile, the effect of contact is improved due to the fixed electrode alternating by the elastic structure, and the contact time is effectively prolonged. 

The simulation and experimental results are listed as Table 2. First, the comparative study of the response time of all models shows that the results of the simulation and experiment show a decreasing trend. The experimental measurement value of response time is longer than the simulation, as shown in Table 2, and is due to the air damping being ignored in the simulation and the pulse of the load applied in the test being wider than the simulation value. Figure 13 indicates that the pulse widths of the acceleration applied to models 1, 2, 3, 4, and 5 are about 8, 3, 2, 3, and 2 ms, respectively. As the pulse width of the applied acceleration is an important factor affecting the response of the movable electrode, the test value is larger than the simulation. Secondly, Table 2 shows that the measured contact time is much larger than the simulation results at a low threshold. In addition, the low threshold (20 g) acceleration load frequency of the tested equipment in this experiment is too low and the pulse is wide, so this is the main reason why the contact time of the experimental test is longer than the simulation result. The contact time of model 2 and 3 shown in Table 2 indicates that the test results are much lower than the simulation, the main reason being that the elastic recovery force of the fixed electrode is an important factor affecting the contact time when the threshold is increased. The larger the threshold, the greater the overload impacted on the fixed electrode during the test, so the movable electrode can obtain a greater elastic recovery force and promote the movable electrode to quickly return to the equilibrium position. Therefore, the contact time of model 2 and model 3 is very short. This is also an important reason why the fixed electrode of the high-G inertial switch needs to use the spring. However, the load applied to the model is an ideal acceleration without overload, and the elastic recovery force of the movable electrode mainly comes from the suspension spring of the movable electrode, so the simulation contact times of model 2 and model 3 are larger than the actual measured value.

In other designs (model 4 and model 5), Table 2 shows that the contact time can be effectively extended owing to the spring fixed electrode. The contact time measured by the experiment is longer than that from the simulation because the friction resistance between electrodes has the effect of prolonging the contact time during the actual test. Thirdly, Table 2 shows that there are differences between the test threshold and the design value, the main reason being that there are some errors between the actual structural parameters of the prototype devices and the designed models. 

## 7. Conclusions

To investigate the dynamic contact process of the inertial switch, a simple and effective experimental design is presented in this paper. An inertial switch with increasing threshold is proposed, and especially, an elastic fixed electrode is proposed for the inertial switch with a high threshold acceleration. The basic working principle and simulation analysis method are used to analyze the dynamic contact process. The results of the analysis indicate that the dynamic contact process is affected by the increasing threshold, which is manifested in the fact that the contact time and response time become shorter with increasing threshold. This is because the frequency of acceleration load increases with the increment in the threshold, and the response frequency of the movable electrode also increases, resulting in a shorter contact time and response time. In addition, the analysis results also show that the contact effect of the high-G inertial switch can be improved by the elastic fixed electrode.

The prototypes are successfully fabricated by surface micromachining technology and are tested by a dropping hammer system. The test results verified the theoretical and simulation results, where the contact time of the inertial switch with a threshold of 580 g is extended to 100 μs. 

By analyzing and testing the dynamic contact process of the inertial switch with different thresholds, the law that the contact time and response time of the inertial switch decrease with increasing threshold is explained, and the solution to extend the contact time of the inertial switch with a high-G value is proposed, which can provide new ideas for other similar devices.

## Figures and Tables

**Table 1:** Main geometric parameters of designed inertial switch.

Models	Proof Mass (μm)		Gap (μm)	Movable Electrode (μm)		Fixed Electrode (μm)	
	*t*	*l*	*g*	*w*	*d*_1_	*d*_2_	*w*_1_
Model 1	60	1200	20	12	10	20	-
Model 2	60	900	20	12	10	20	-
Model 3	60	700	20	15	10	20	-
Model 4	60	900	20	12	10	20	25
Model 5	60	700	20	15	10	20	30

**Table 2:** Comparative analysis of analytical, simulation, and experimental results.

Devices	Model 1			Model 2			Model 3			Model 4			Model 5		
Parameters	a_th_ (g)	t_re_ (µs)	t_con_ (µs)	a_th_ (g)	t_re_ (µs)	t_con_ (µs)	a_th_ (g)	t_re_ (µs)	t_con_ (µs)	a_th_ (g)	t_re_ (µs)	t_con_ (µs)	a_th_ (g)	t_re_ (µs)	t_con_ (µs)
Unit															
**Simulation**	20	1000	65	280	280	40	580	220	35	280	280	50	600	220	50
**Test**	20	4000	950	295	700	15	580	400	5	305	700	100	580	400	100
